# Supplementary material for: Myzus persicae Management through Combined Use of Beneficial Insects and Thiacloprid in Pepper Seedlings
Source: Insects. 2021 Sep 3;12(9):791. doi: 10.3390/insects12090791 (PMC8464916; doi:10.3390/insects12090791)

Table S1 Concentration range used in the acute toxicity determination the aphids and the natural enemies.

Pesticides	Insects	Concentration range (mg a.i.·L^{-1})	doses
**Imidacloprid**	*M. persicae*	0.007-700	6
	H. axyridis	0.011-7.00	5
	A. aphidimyza	0.022-14.00	5
**Nitenpyram**	*M. persicae*	0.001-100.00	6
	H. axyridis	6.25-100	5
	A. aphidimyza	0.0032-2.00	5
**Acetamiprid**	*M. persicae*	1.95-500.00	5
	H. axyridis	0.0063-2.00	5
	A. aphidimyza	0.0064-4.00	5
**Thiacloprid**	*M. persicae*	0.0002-20.00	6
	H. axyridis	0.125-4.00	6
	A. aphidimyza	0.02-200.00	5
**Thiamethoxam**	*M. persicae*	0.0025-250.00	6
	H. axyridis	0.04-25.00	5
	A. aphidimyza	0.008-5.00	5
**Clothianidin**	*M. persicae*	0.002-200.00	6
	H. axyridis	0.0032-2.00	5
	A. aphidimyza	0.0064-4.00	5
**Dinotefuran**	*M. persicae*	0.128-400.00	6
	H. axyridis	0.0064-4.00	5
	A. aphidimyza	0.0064-4.00	5
**Flupyradifurone**	*M. persicae*	0.002-200.00	6
	H. axyridis	0.50-8.00	5
	A. aphidimyza	0.0026-8.00	6

Table S2 Concentration range used in the acute toxicity determination of *B. terrestris.*

Pesticides	Insects	Concentration range (μg a.i.·bee^{-11})	doses
**Nitenpyram**	*B. terrestris*	75.00-1200.00	5
**Thiacloprid**	*B. terrestris*	125.00-2000.00	5

Figure S1 Temperature recorded every hour during the tests. Where L = low temperature and H= high temperature.

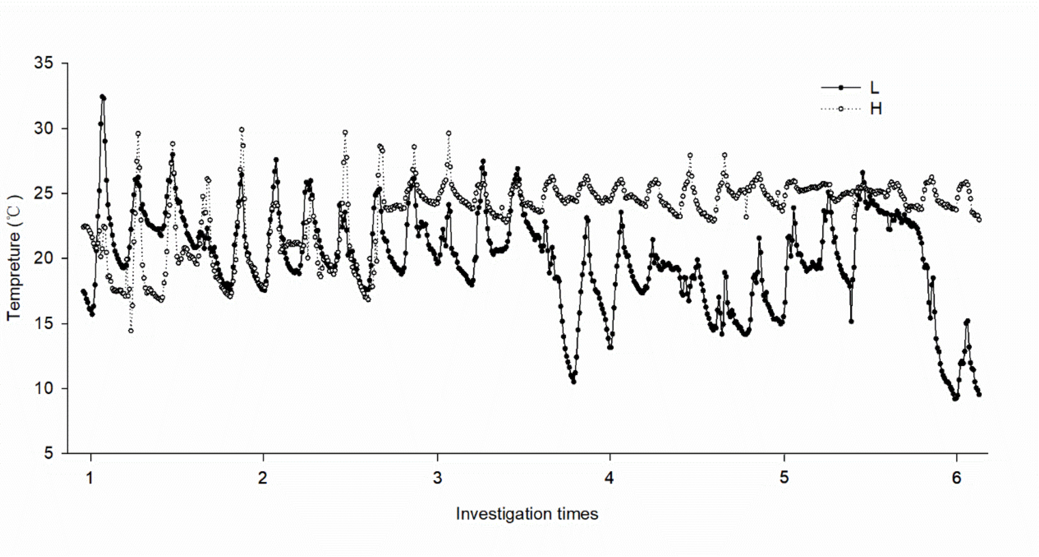

Supplement: Supplementary file 1 [file insects-12-00791-s001.zip › insects-1313923-supplementary.pdf]
